# Comprehensive phenotyping of *RFC1* -related disorder: integrating electrophysiological, brain imaging, and otoneurological data in deep phenotyping

**DOI:** 10.1055/s-0045-1811723

**Published:** 2025-10-27

**Authors:** André Aires Fernandes, Pedro L. Alexandre, Sofia Vedor, Rita Figueiredo, Pedro Marques, Luís Braz

**Affiliations:** 1 Unidade Local de Saúde de São João, Serviço de Neurologia, Porto, Portugal.; 2 Universidade do Porto, Faculdade de Medicina, Departamento de Neurociências Clínicas e Saúde Mental, Porto, Portugal.; 3 Unidade Local de Saúde de São João, Serviço de Otorrinolaringologia, Porto, Portugal.; 4 Universidade do Porto, Faculdade de Medicina, Departamento de Cirurgia e Fisiologia, Unidade de Otorrinolaringologia, Porto, Portugal.; 5 Unidade Local de Saúde de São João, Serviço de Neuroradiologia, Porto, Portugal.

**Keywords:** Cerebellar Ataxia, Peripheral Nervous System Diseases, Vestibular Diseases, Ataxia, Neurologic Examination

## Abstract

**Background:**

The syndrome defined by cerebellar ataxia, neuropathy, and vestibular areflexia (CANVAS) has been previously described as a cause of late-onset ataxia. With the discovery of biallelic expansion in the replication factor C subunit 1 ( *RFC1* ) gene as its underlying genetic cause, this syndrome and the broader gene disease became more clinically heterogeneous and one of the most common genetic causes of ataxia in adults.

**Objective:**

To characterize the phenotypic spectrum of *RFC1* expansion using a multidisciplinary approach combining neurological, otoneurological, and neuroimaging assessments.

**Methods:**

A retrospective cohort study comprising patients with a genetically confirmed diagnosis of biallelic *RFC1* repeat expansions was conducted. Data related to neurological examination, video head impulse test (vHIT), caloric tests, posturography, electromyography/nerve conduction studies and brain magnetic resonance imaging (MRI) were considered.

**Results:**

We included 15 patients, of whom 10 (66.7%) presented with the complete clinical triad. At neurological examination, 13 patients showed signs of peripheral neuropathy. Cerebellar dysfunction was observed in 12, whereas postural instability was seen in 11. Electromyography/nervous conduction studies revealed peripheral neuropathy in all of the cases, while bilateral vestibular dysfunction was confirmed in approximately half of them. The mean balance values from the posturography were lower in the majority ( *n* = 14). In the imaging assessment ( *n* = 11), 6 patients displayed significant vermian atrophy, predominantly in the anterior/dorsal regions, while the other 5 patients showed moderate atrophy.

**Conclusion:**

This study underscores the clinical importance of comprehensive phenotyping and multimodal diagnostic approaches—including neurological, otoneurological, electrophysiological, and imaging assessments—in enhancing diagnostic precision, especially when neurological examination findings are inconclusive or in atypical/incomplete clinical presentations.

## INTRODUCTION

Cerebellar ataxia, neuropathy, and vestibular areflexia syndrome (CANVAS) has been characterized as a form of slowly progressive, late-onset ataxia. 1 2 3 This condition arises from any combination of impairments in the vestibular system, peripheral nerves (dorsal root ganglia), and cerebellum. 2 Although cases of combined ataxia and vestibulopathy were first reported in the 1990s, CANVAS was only formally identified as a distinct clinical entity in 2011. 4 5 In 2019, Cortese et al. discovered that biallelic expansions in the replication factor C subunit 1 ( *RFC1* ) gene are the genetic cause of this syndrome, a finding that revolutionized the diagnostic approach to this autosomal recessive syndrome. 6 This discovery allows for diagnosis even in the absence of the complete classic triad, thereby redefining the condition as an *RFC1* -related disorder. 6 7

The phenotypic spectrum associated with biallelic intronic repeat expansions in *RFC1* includes a range of symptoms such as typical cerebellar ataxia, sensory neuropathy, and vestibular areflexia syndrome. Also, subclinical impairments in cerebellar, sensory, and vestibular functions; autonomic dysfunction; chronic cough; and more limited phenotypes primarily affecting one of the systems involved in balance control. Additional neurological features reported within the *RFC1* spectrum include motor neuropathy, hyperkinetic movement disorders, Parkinsonism, rapid eye movement (REM) and non-REM sleep disorders, and cranial neuropathy. 8 9 The shift from a predominantly clinical-based diagnosis to genetic testing has facilitated more accurate diagnoses, positioning *RFC1* -related disorders as a major genetic cause of adult-onset cerebellar ataxia and a significant contributor to idiopathic sensory neuropathy. 2 7 10

Despite recent genetic advancements, neurological examination remains fundamental for identifying symptomatic individuals potentially affected by this condition, as it can reveal vestibular, cerebellar, and sensory impairments. Indeed, the majority of previous studies have predominantly focused on neurological characterization. Nevertheless, the combined use of vestibular and posturographic complementary diagnostic tools could provide valuable additional information, both during the initial assessment of patients with ataxia and throughout follow-up. This multimodal approach may contribute to a more comprehensive clinical characterization, enhancing diagnostic precision and supporting more personalized care.

Our objective is to enhance the understanding of the phenotypic spectrum directly caused by *RFC1* expansion through an interdisciplinary approach, including neurological, otoneurological, and neuroimaging evaluations.

## METHODS

### Patient data

Retrospective analysis of a cohort of patients followed in a tertiary center followed at a tertiary center outpatient neuromuscular clinic between January 2016 and 2025. All the individuals included had the biallelic pentanucleotide repeat expansion in the gene encoding *RFC1* . Patients' records were retrospectively evaluated, including family history, clinical data, evidence of peripheral neuropathy, and cerebellar and/or vestibular dysfunction. All patients were clinically classified according to three main axes: peripheral neuropathy, vestibular impairment and cerebellar dysfunction.

Sensory neuropathy/neuronopathy was inferred by the presence of related symptoms (loss of feeling, paresthesia, dysesthesia, neuropathic pain) and/or abnormal sensory exam, including ataxia. Abnormal head impulse test (HIT) and/or oscillopsia were used to access the presence of vestibulopathy. Cerebellar dysfunction was deduced from neurological examination findings, such as cerebellar dysarthria, dysmetria, and/or abnormal eye movements (nystagmus, dysmetric saccades, impaired ocular pursuit).

Additionally, vestibular impairment was further evaluated through the video head impulse test (VHIT), caloric test and posturography. Data from electromyography/nerve conduction studies and brain magnetic resonance imaging (MRI) were also collected.

### Pentanucleotide repeat expansion testing for *RFC1*

Biallelic AAGGG repeat expansion in *RFC1* was searched by repeat and flanking-primed polymerase chain reaction (PCR). 6 11 Expansions of additional likely nonpathogenic repeat configurations, ACAGG repeat expansions, and truncating variants were not searched.

### Electrodiagnostic testing

Nerve conduction studies were conducted using standard techniques, encompassing motor nerves (median, ulnar, tibial, peroneal) and antidromic sensory nerves (median, ulnar, radial, sural, and/or superficial peroneal). 12 13 Concentric needle electromyography was performed based on clinical discretion. All electrophysiological assessments were performed using a Keypoint G4 (Natus Medical Inc.) system. A qualitative analysis was provided based on the comprehensive findings. Sensory neuronopathy was defined by diffusely reduced or absent sensory nerve action potential (SNAP) in a nonlength-dependent pattern (i.e., both upper and lower limbs, not explained by entrapment neuropathies) + no more that 1 abnormal motor nerve conduction study. Axonal polyneuropathy was defined by reduced or absent SNAPs +- compound motor action potential (CMAP) following a length-dependent pattern (i.e., distal lower limbs more affected than distal upper limbs). 13 14

### Vestibular testing

The Otometrics ICS Impulse (Natus Medical Inc.) system was used for vHIT, documenting gains across all six semicircular canals as well as overt and covert saccades. Videonystagmography (VNG) was recorded using the VNG Ulmer SYNAPSIS (Inventis Inc.) system. Bithermal caloric tests were conducted by stimulating the external auditory canals with cold (30°C) and warm (44°C) water.

Bilateral vestibulopathy was defined according to the diagnostic criteria consensus of the Bárány Society for bilateral vestibulopathy, published in 2017. 15 Specifically, bilateral vestibulopathy was diagnosed if the horizontal canal angular vestibulo-ocular reflex gain was less than 0.6 on vHIT, or if the sum of the bithermal maximum peak slow phase velocities on each side was less than 6°/sec in the caloric test. 15

### Posturography

Computerized dynamic posturography was conducted using the NeuroCom EquiTest (Natus Medical Inc.) system. The results of the sensory organization test (SOT) and the Limits of Stability were documented.

### Magnetic resonance imaging

Brain MRI examinations were conducted using standard clinical protocols on 1.5T and 3T scanners. The minimum protocol included coronal T2-weighted, 3D fluid-attenuated inversion recovery (FLAIR), and 3D T1. All images were independently reviewed by two experienced neuroradiologists; the 3D T1 images were evaluated to assess cerebellar atrophy, by applying a qualitative analysis of the potentially involved regions.

### Statistics

Statistical analysis was performed using the IBM SPSS Statistics for Windows (IBM Corp.), version 27.0. Data were expressed as means ± standard deviation (SD). Normality of data was assessed using the Kolmogorov-Smirnov test. Group comparisons were made using the χ ^2^ test, Mann-Whitney U test, and Student's *t* -test, as appropriate ( *p* -values < 0.05 were deemed statistically significant).

Missing data were explicitly reported at each relevant point throughout the manuscript. Analyses were conducted based on available data, and cases with missing values for specific variables were excluded from the corresponding analyses, without imputation.

### Human ethics and consent to participate

The present study was conducted according to the principles of the Helsinki declaration and patient written consent was obtained. This study was approved by the Ethics committee of Unidade Local de Saúde de São João (n°320/22).

## RESULTS

Among the 15 patients harboring biallelic AAGGG repeat expansions in the *RFC1* gene, 8 (53.3%) were male. Demographic and clinical characteristics are summarized in Table 1 . The cohort had a mean age of 65.8 ± 12.8 years, with a mean age at clinical onset of 53.9 ± 12.0 years. Imbalance was reported as the initial neurological symptom in 11 (73.3%) subjects. Family history suggestive of inherited neuropathy was noted in 8 (53.3%). Chronic idiopathic cough was present in 8 patients (53.3%). At the last assessment, 10 patients (66.6%) exhibited the classical triad of symptoms. There were complaints of liquid dysphagia from 6 (40.0%). Additionally, other related symptoms/complaints were evaluated, including constipation ( *n* = 2), sexual dysfunction ( *n* = 1), and urinary incontinence ( *n* = 1).

**Table 1:** Demographic and clinical data, including referred CANVAS-related symptoms data (n = 15)

**Gender (male)**	8 (53.3%)
**Family history (yes)**	8 (53.3%)
**Mean age at examination**	65.8 ± 12.8
**Age at onset**	53.9 ± 12.0
**Age at diagnosis**	65.2 ± 12.6
**Mean disease duration (years)**	11.2 ± 8.1
**First neurological symptom**	
Imbalance	11(73.3%)
Neuropathic symptoms	3 (20.0%)
Dysarthria	1 (6.7%)
**Postural imbalance**	11 (77.3%)
**Gait abnormality**	13 (86.7%)
Walks without help	8
Walks with unilateral assistance	5
Wheelchair	0
**Dysphagia (yes)**	6 (40.0%)
**Chronic idiopathic cough (yes)**	8 (53.3%)
**Other relevant symptoms/conditions**	6 (40.0%)
Constipation	2
Sexual dysfunction	1
Urinary incontinence	1
Restless legs syndrome	1
Optic neuropathy	1

Abbreviation: CANVAS, cerebellar ataxia, neuropathy and vestibular areflexia.

All patients underwent evaluation by a senior neurologist specializing in neuromuscular disorders, and findings from previous neurological examinations are summarized in Table 2 . At the latest assessment, 13 patients (86.7%) presented with distal length-dependent sensory deficits, including 2 with concurrent distal motor deficits. The majority ( *n* = 10, 66.7%) had bilaterally abnormal HIT, and 6 (40.0%) patients exhibited cerebellar ocular motor signs, such as downbeat nystagmus ( *n* = 3), simultaneous jerky smooth pursuit and saccadic dysmetria ( *n* = 1), as well as alternant strabismus ( *n* = 1).

**Table 2:** Neurologic examination findings (n = 15)

		n (%)
**Completed clinical triad**		10 (66.6)
**Pathologic HIT**		11 (73.3)
Unilateral		1
Bilateral		10
**Sensory examination (abnormal** **pinprick distal limbs)**		13 (86.7)
Upper limbs		6
Lower limbs		11
**Ataxia**	Axial/gait	13
	Limb	0
**Dysarthria (yes)**		5 (33.3)
**Abnormal eye movements**		6 (40.0)
Downbeat nystagmus		3
Jerky smooth pursuit + saccadic dysmetria		1
Alternant strabismus		1
Skew deviation		1
**Deep tendon reflexes**		
**Ankle**	Normal	5 (33.3)
	Reduced	4 (26.7)
	Absent	6 (40.0)
**Knee**	Hyperactive	2 (13.3)
	Normal	6 (40.0)
	Reduced	4 (26.7)
	Absent	3 (20.0)

Abbreviation: HIT, head impulse testing.

Skew deviation on the cover test was noted in 1 patient. Knee reflexes were elicitable in 6, reduced or absent in 7, and brisk in 2 patients. Ankle reflexes were more frequently reduced or absent ( *n* = 10), but preserved reflexes were observed in 5 patients. Cerebellar ataxia was evident in 13 (86.7%), with only 5 (33.3%) requiring unilateral assistance despite exhibiting ataxic gait. Those requiring assistance tended to have a longer disease duration (14.0 ± 6.51 vs. 9.8 ± 8.69 months, *p* = 0.360). Dysphagia was noted in 6 patients (40.0%), being positively associated with disease duration ( *p* = 0.003). No significant differences were observed between patients presenting with and without the complete triad regarding age at disease onset, disease duration, and time to loss of independent gait ( *p* = 0.963, 0.435, and 0.600, respectively).

Electrodiagnostic assessments ( Table 3 ) identified peripheral neuropathy in all subjects. Sensory axonal polyneuropathy was found in 8 (53.3%) patients, sensory neuronopathy in 3 (20.0%) and motor and sensory axonal polyneuropathy in 4 (26.7%).

**Table 3:** Electromyography/nerve conduction studies (n = 15)

	n (%)
**Abnormal**	15 (100)
Sensory axonal neuropathy	8 (53.3)
Moderate	1
Severe	7
Sensory neuronopathy*	3 (20)
Moderate	1
Severe	2
Motor and sensory axonal neuropathy	4 (26.7)
Severe sensory and mild motor impairment	4

**Figure 1:**
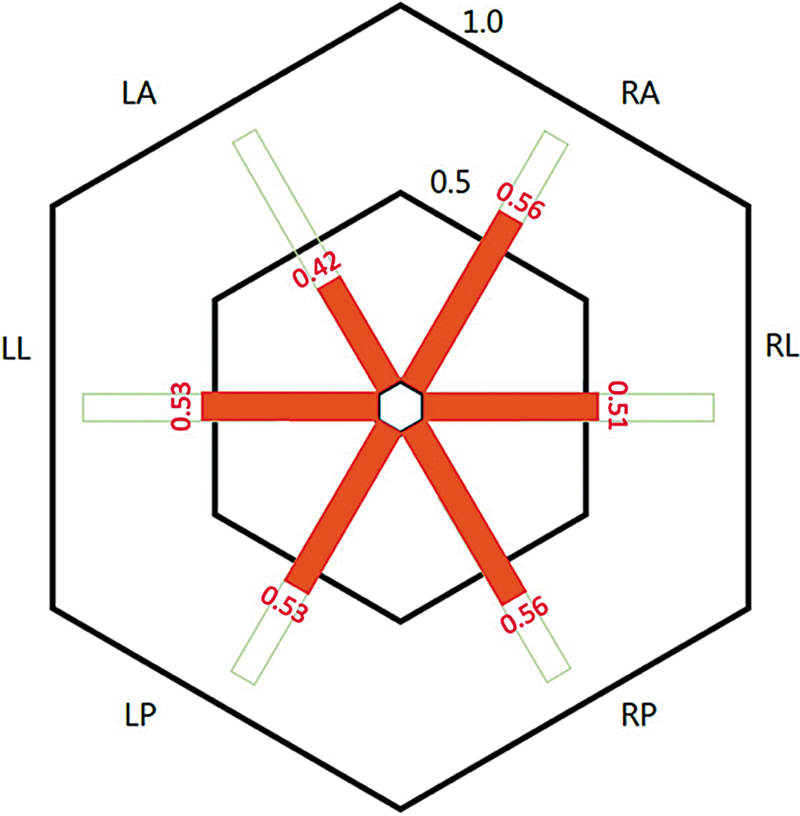
Notes: LA left anterior SSC, LL left lateral SCC, LP left posterior SCC, RA right anterior RL right lateral SCC, RP right posterior SCC. VHIT gains on the six semicircular canals.

Vestibular function tests were performed in all patients ( Table 4 ). Abnormalities in vHIT were noted in 11 patients (73.3%), with 8 (53.3%) meeting criteria for bilateral vestibular hypofunction and 3 (20.0%) displaying unilateral involvement. All of the 6 patients diagnosed with bilateral vestibulopathy on caloric testing also exhibited bilateral hypofunction on vHIT. Mean gains on all six canals during the test were reduced ( Figure 1 ), and overt and covert saccades were frequently observed.

**Table 4:** Video head impulse test and caloric test results*

Patient	Right lateral SCC gain	Left lateral SCC gain	Right lateral SCC saccades (covert/overt)	Left lateral SCC saccades (covert/overt)	Caloric response in the most responsive side (°/s)
1	**0.41**	**0.36**	0/1 ^2^	0/1	6.6
2	**0.11**	**0.18**	1/1	1/1	**4.7**
3	**0.31**	**0.27**	1/1	1/1	**1.5**
4	**0.51**	0,79	1/1	1/1	9.6
5	**0.03**	**0.09**	1/1	1/1	**1.2**
6	0,92	0,89	1/1	0/1	60
7	1.02	1.00	1/1	1/1	16.9
8	1.01	0,90	0/1	1/1	22.1
9	**0.45**	**0.43**	1/1	0/1	30.5
10	**0.33**	**0.32**	1/1	1/1	**4.4**
11	0.74	**0.49**	1/1	1/1	missing data
12	1.09	1,05	0/0	0/0	20.9
13	**0.32**	**0.34**	1/1	0/1	**4.1**
14	**0.56**	0.61	1/1	1/1	12.4
15	**0.04**	**0.17**	1/1	1/1	**4.3**

Abbreviation: SCC, semicircular canal.

Notes: *Values in bold are considered abnormal. According to the Bárány society criteria, 8 patients (53.3%) were classified as having bilateral vestibulopathy; 0–saccade present, 1–saccade absent.

**Figure 2:**
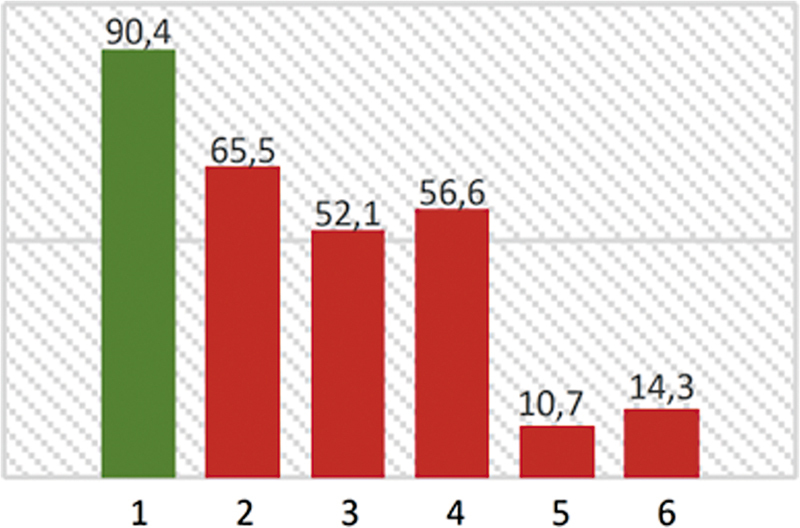
Sensory organization test average results (conditions 1 to 6).

Regarding posturography ( Figure 2 ), global values of the SOT were reduced in the majority ( *n* = 14; 93.3%), with average results notably decreased in conditions 2 to 6. Multisensory deficits and visual dependence patterns were commonly observed. End-point (EPE) and maximum (MXE) excursions regions of stability were globally reduced.

**Figure 3:**
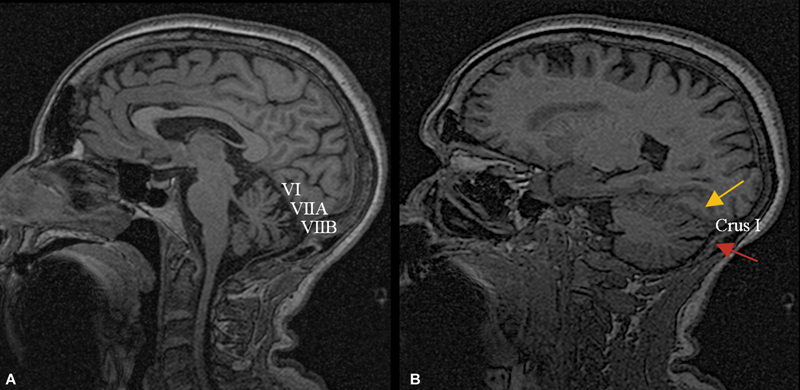
Brain MRI: 3D sagittal T1-weighted image illustrating a characteristic pattern of cerebellar atrophy on CANVAS. Atrophy of ( **A** ) the anterior and dorsal cerebellar vermis, between the primary and prepyramidal fissures, corresponding to VI, VIIA and VIIB lobules; ( **B** ) and lateral hemispheric, predominantly affecting the Crus I lobule, which is translated by a slight enlargement of the posterosuperior (gray arrow) and horizontal (red arrow) fissures.

Brain MRI data ( Table 5 ) were available for 11 patients (73.3%). Of these, 8 were scanned using a 1.5T system and 3 using a 3T system. As this was a retrospective study, imaging was acquired through routine clinical protocols without predefined harmonization between scanners. Nevertheless, image quality was deemed sufficient in all cases to allow for qualitative assessment of cerebellar atrophy. Marked vermian atrophy with anterior/dorsal predominance was observed in 6 (54.5%) patients, while moderate atrophy was noted in the remaining 5. The degree of atrophy in Crus I varied widely, with severe patterns in 3 patients, moderate in another 3, mild in 3, and nonvaluable in 2 patients (18.2%). These patterns of cerebellar atrophy distribution are illustrated in Figure 3 .

**Table 5:** Qualitative MRI assessment of cerebellar atrophy (n = 11)

		n (%)
Vermian atrophy (+ anterior/dorsal)	Moderate	5
	Severe	6
Cerebellar hemisphere atrophy (+ crus I)	Nonvaluable	2
	Mild	3
	Moderate	3
	Severe	3

Abbreviation: MRI, magnetic resonance imaging.

## DISCUSSION

Advancements in understanding the *RFC1* -related disorder have markedly accelerated in recent years, particularly following the gene's discovery in 2019. 2 Although the precise pathogenic mechanisms underlying biallelic *RFC1* expansions remain elusive, the ability to achieve a molecular genetic diagnosis has positioned the disorder as one of the most prevalent late-onset ataxias. 2 10 This molecular insight has significantly improved diagnostic sensitivity and specificity, enabling earlier identification of patients who may present with partial manifestations of the triad. 2 3

Notably, peripheral neuropathy was universally identified in electrodiagnostic studies and could be detected on physical examination in the majority (86.7%) of patients. Clinical cerebellar dysfunction was present in a substantial majority (86.7%) of patients, with corresponding imaging evidence of atrophy observed in all examined brain MRIs. Similarly, 73.3% of patients exhibited vestibular impairment, confirmed either clinically or through vHIT/caloric testing, although only 8 (53.3%) cases met the criteria for bilateral vestibulopathy. The observed concordance rate between HIT and vHIT was 73.3%.

Dynamic posturography offers a more objective quantification of the balance and how patients use its sensory systems (proprioceptive, visual, and vestibular) to maintain stability. Instability is a usual finding in patients with *RFC1* -related disorder, as was observed in the series. In fact, dynamic posturography revealed reduced global values in the SOT in the majority (93.3%) of patients. Noteworthy, multisensory deficits and patterns of visual dependence were frequently observed within the cohort. As suggested by others, we advocate that posturographic studies should be a part of the evaluation of patients with instability of any origin. 16

From a clinical standpoint, the classical triad of *RFC1* -related disorder symptoms was evident in only 10 patients (66.6%). However, this number increased to 11 (73.3%) upon comprehensive neurological examination and to 12 (80.0%) when including ancillary exams. As previously reported, *RFC1* -related disorder manifests as a slowly progressive condition, with the majority of patients exhibiting ataxic gait, although only 5 required unilateral assistance. In the present study, individuals requiring assistance exhibited a tendency toward longer disease duration, although this difference did not reach statistical significance. Notably, dysphagia was identified in 40.0% of patients and showed a significant positive association with disease duration. This association underscores the potential utility of dysphagia as a surrogate indicator of disease severity and cerebellar dysfunction in this population.

None of the patients required percutaneous endoscopic gastrostomy due to manageable mild to moderate dysphagia using behavioral measures and thickeners.

Our findings are consistent with those of a larger clinical cohort of *RFC1* -related disorder patients from the UK, albeit without inclusion of posturography. 7 Recent studies have predominantly focused on clinical neurological examination findings. Integration of otoneurological assessments, particularly vHIT, caloric tests, and posturography, provides a comprehensive evaluation of vestibular dysfunction. We advocate for a multimodal phenotyping approach essential for elucidating the full spectrum of phenotypes associated with *RFC1* expansion. Another strength of our study lies in the cohort's homogeneity, comprising exclusively positive patients, in contrast to clinical-defined *RFC1* -related disorder cohorts. 17 18 19 20

A notable limitation is the small size of our cohort, reflecting the rarity of the disease and its recent recognition, potentially leading to underdiagnosis. Nonetheless, due to the uncommon nature and recent characterization of *RFC1* disease, our findings contribute novel insights. Additionally, the length of pentanucleotide repeat expansions was not quantified, precluding assessment of potential associations with disease severity and age of onset, as suggested in recent literature. 21 A small number of cases with typical *RFC1* -related disorder do not carry the common biallelic repeat expansion. 22 However, additional repeat configurations and truncating variants were not searched.

Concerning neuroimaging, quantitative or semiquantitative MRI rating scales were not employed in this study, which may have limited the objective characterization of imaging findings. Finally, the retrospective nature of our study represents a significant limitation, dependent on the quality of clinical records.

The discovery of the pathogenic role of *RFC1* expansions has broadened the clinical spectrum of CANVAS, enabling diagnosis beyond the classic triad. This study highlights the value of detailed phenotyping and multimodal diagnostic integration in refining the characterization of *RFC1* -related disorders and advancing understanding of their variable presentations and progression.
